# The planning and establishment of a sample preparation laboratory for drug discovery

**DOI:** 10.1155/S1463924600000316

**Published:** 2000

**Authors:** Claude Dufresne

**Affiliations:** Natural Products Drug Discovery Automation & Informatics Group Merck Research Laboratories R80Y-340, 126 E. Lincoln Ave Rahway NJ 07065 USA

## Abstract

Nature has always been a productive source of new drugs. With the advent of high-throughput screening, it has now become possible to rapidly screen large sample collections. In addition to seeking greater diversity from natural product sources (micro-organisms, plants, etc.), fractionation of the crude extracts prior to screening is becoming a more important part of our efforts. As sample preparation protocols become more involved, automation can help to achieve and maintain a desired sample throughput. To address the needs of our screening program, two robotic systems were designed. The first system processes crude extracts all the way to 96-well plates, containing solutions suitable for screening in biological and biochemical assays. The system can dissolve crude extracts, fractionate them on solid-phase extraction cartridges, dry and weigh each fraction, re-dissolve them to a known concentration, and prepare mother plates. The second system replicates mother plates into a number of daughter plates.


Journal of Automated Methods & Management in Chemistry, Vol. 22, No. 6 (November–December 2000) pp. 175–179

### The planning and establishment of a sample preparation laboratory for drug discovery

**Claude Dufresne**

*Natural Products Drug Discovery, Automation & Informatics Group, Merck Research Laboratories, R80Y-340, 126 E. Lincoln Ave, Rahway, NJ 07065, USA*

*Nature has always been a productive source of new drugs. With the advent of high-throughput screening, it has now become possible to rapidly screen large sample collections. In addition to seeking greater diversity from natural product sources (microorganisms, plants, etc.), fractionation of the crude extracts prior to screening is becoming a more important part of our e orts. As sample preparation protocols become more involved, automation can help to achieve and maintain a desired sample throughput. To address the needs of our screening program, two robotic systems were designed. The first system processes crude extracts all the way to 96-well plates, containing solutions suitable for screening in biological and biochemical assays. The system can dissolve crude extracts, fractionate them on solid-phase extraction cartridges, dry and weigh each fraction, re-dissolve them to a known concentration, and prepare mother plates. The second system replicates mother plates into a number of daughter plates.*

### Introduction

Nature is unmatched in its ability to create small organic molecules enriched with biological potency and structural diversity. Chemical diversity is currently sought through biodiversity of natural product-producing sources: microorganisms (prokaryotes and eukaryotes), plants, marine macroorganisms, insects. From 1983 to 1994, of the 520 new Food and Drug Administration approved drugs, nearly 40% were derived from natural products (30 are unmodified natural products, 127 are semi-synthetic derivatives, and 46 are synthetic compounds modelled after a natural product parent) [1].

In screening natural sources, at the minimum, a crude extract is generated. This is usually accomplished by treating the source with an organic solvent (EtOAc, MEK, etc). Crude extracts can then be partitioned between various solvents to minimize interferences (fatty acids, salts, etc). In addition to seeking greater diversity of samples from the intrinsic diversity of natural products sources, fractionation of the crude extracts prior to screening is becoming a more important part of our efforts. Fractionated samples contain a smaller number of individual compounds and are less likely to give erroneous assay results (false positives and/or masked activities). Fractionation thus improves the quality of screening samples. However, increased sample processing increases the possibilities of degrading unstable compounds, as well as 'losing' others owing to irreversible binding to chromatography supports. Furthermore, processing time, whether using manpower or automated equipment is costly. The most important downside of fractionation as part of a sample preparation process, is the increase in costs resulting from the increased number of samples. However, with the advent of high-throughput screening (HTS) and higher density formats, screening increasingly larger sample collections is becoming less of a problem.

To partly address the needs of our screening program, automated systems were designed. Processes were divided between two robotic systems. The first system, the sample preparation system, processes samples from crude extracts all the way to 96-well mother plates, containing solutions suitable for screening assays. The second system replicates mother plates into a number of daughter plates. The process of fractionating samples using solid-phase extraction (SPE) will not be covered in this presentation.

### General considerations

Following the initial extraction, samples are usually handled as solutions in tubes or vials. When drying is performed, tubes are preferred for their round shaped bottom. Volumes vary from a few millilitres to several hundred millilitres. The end product of the first system had to be deep well mother plates. For convenience and flexibility, we decided to use racks of tubes that would have the same outside dimensions as that of a 96-well plate. In that way, the same robot gripper could be used for both plates and racks of tubes. Furthermore, the same storage devices, such as carousels, could be used for both types of container. After looking at various possibilities, we chose 16 mm ID tubes. With that diameter, racks of 24 tubes ($4 \times 6$) can have the required footprint. In addition, these racks would map nicely to the four quadrants of a 96-well plate. Even though we currently use only 80 of the 96 wells, as a company standard for sample distribution, designing the racks to map to full plates would allow us to avoid obsolescence as long as the 96-well format remained in existence. We would therefore use only 20 of the 24 tube locations, but would design the equipment to be able to use all positions. Tube height was selected as 100 mm. Even though higher tubes (150 mm) provided for additional volume capacity (15 ml vs 10 ml), they had several problems: drying was more problematic in the longer tubes, storage density was decreased, and access with liquid handlers was more difficult (clearance, reach). Our processes were thus adapted to a 10 ml working volume.

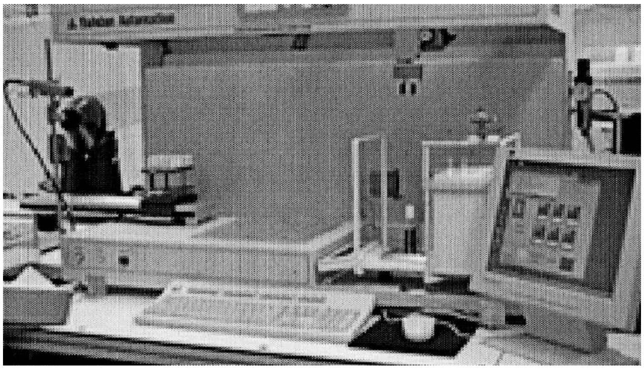

*Figure 1. Bohdan weigher workstation.*

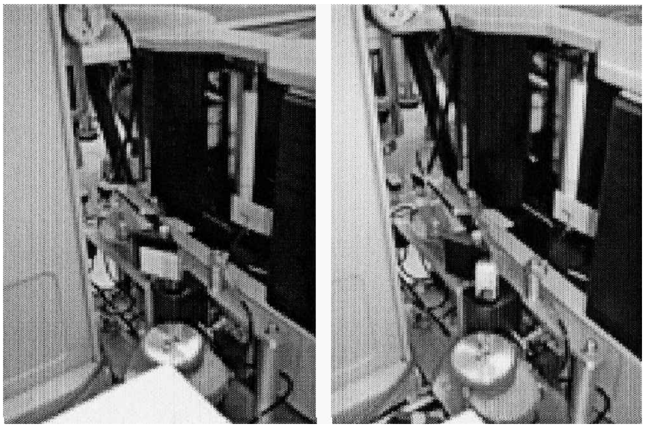

*Figure 2. Label application.*

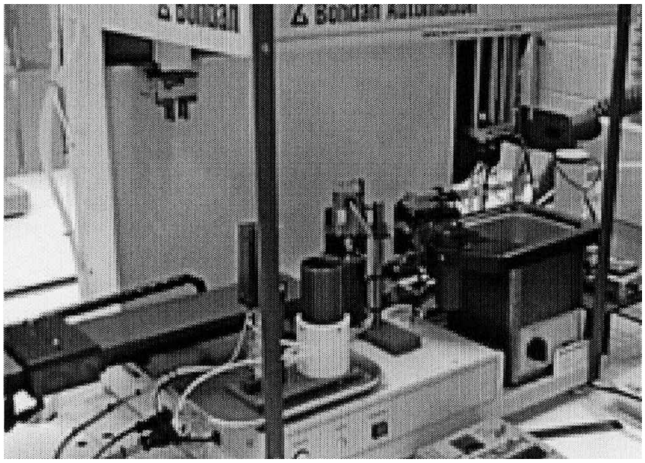

*Figure 3. Bohdan dissolution workstation.*

### Sample preparation system

It was decided to structure our automated system around the concept of using workstations where samples are transported in groups as opposed to individually. Furthermore, for maximum flexibility in end-user interactions, each workstation should be usable in both a manual and an automated mode. In manual mode, the user would place racks of samples on the workstation deck, while in automated mode, the robot would bring the samples. This dual mode can be readily achieved by using workstations that can be accessed from the rear by the robot. The workstations then face the user as they normally would in stand-alone configurations. Rear access can be accommodated through an opening in the back of the workstation or using a shuttle that extends out of the side of the station (figure 1). It was felt this arrangement would ensure that our work could proceed in the event the full system itself was down due to any one of several reasons.

### *Workstations*

We selected Bohdan Automation, Inc. to build the workstations. The first workstation is a weigher/labeller (figure 1). The workstation can label tubes (Zebra printer) and weigh them using a four-place Mettler balance. A barcode reader is used to positively identify every tube. The manual area can accommodate six racks of tubes (144 tubes), while the side-mounted shuttle, for rear robot access, can hold one rack of 24 tubes.

Label application (figure 2) involves several steps. As the label is printed, it is held on a vacuum transfer plate that pivots to contact a revolving tube. As the tube revolves, the label is applied.

The second workstation (figure 3), for dissolution, incorporates a shower head canula (choice of six solvents), a sonicator bath, a homogenizer probe, and a barcode reader.

The third workstation, for SPE, will be the subject of a separate presentation. The dryer was built in house. Details of a slightly different model, designed for 96-well plates, have been published [2].

A Packard MultiProbe 104 was selected for its ease of integration and macro handling capabilities. Every station (except for the dryer) uses its own control PC. This is necessary to ensure full manual mode operations at any time.

### *Robot*

The A465 robot arm (CRS Automation Solutions, Inc.) was chosen for its payload capability. A metal rack of 24 tubes, filled with solvent, weighs approximately 1 kg which is beyond the safe payload of most small laboratory scale robots. In addition the A465 has a long reach which allows the use of a storage carousel that is 32 inches high. With three out of every four shelves removed from the CRS carousel, we can store any combination of 48 racks of tubes or deep-well plates. The fingers of a standard microtitre plate gripper were modified (figure 4) to be able to grab either a deep-well plate or a rack of tube from the same taught position.

### *Room facility*

With good estimates of system sizes, a laboratory was selected to house the facility. As is almost always the case, there never is a vacant wide open room that is available. We selected a generic laboratory (16′ × 36′), moved out the equipment, and renovated it. New air exhausts were installed, as well as new services (high pressure air,

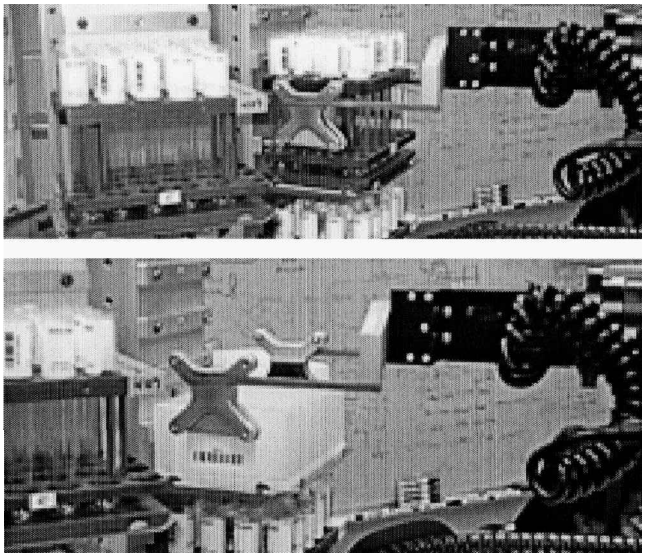

*Figure 4. Dual-mode custom gripper.*

nitrogen, vacuum, water, power, ethernet, phone). A custom table, built with extruded aluminium sections from Item Products and designed to house the robot track below the table surface, was installed. All utilities were built onto the table framework.

*System integration*

The system was integrated around CRS POLARA software (figure 5). POLARA integrated systems currently make use of two integrator provided components: an ActiveX component for user interface (during system configuration and method building) and a RAPL-3 component (for instrument communications and interfacing with the system controller). Since RAPL-3 is designed to work via serial lines, every PC is connected to the robot controller PC using serial lines (we use a Cyclades Z-card). POLARA( also provides built-in support for a PLC (we use Direct PLC) to monitor various sensors.

Custom software was written to integrate the operation of the robot with our corporate registration database (NEXUS). As sets of 20 samples are formed early on in the process, they are grouped in sets of four, assigned a quadrant in ultimate mother plates, registered and assigned new set IDs. As tubes are weighed, the volume of solvent for re-dissolution is calculated, as well as the amount of diluent needed for ‘heavy’ samples. The resulting information (sample amount and concentration) is automatically uploaded to NEXUS.

### Experiences

After over a year of full operation, we have experienced relatively few failure types. In the beginning, the singlemost occurring source of errors was label misapplication. When numerous attempts at adjusting the applicator did not improve outcomes, we elected to purchase pre-labelled tubes. Subsequently, Bohdan modified the applicator mechanism with improved results. The time saving with the purchase of pre-labelled tubes is significant.

With this source of error removed, the next level of failures that became apparent was human errors: mis-

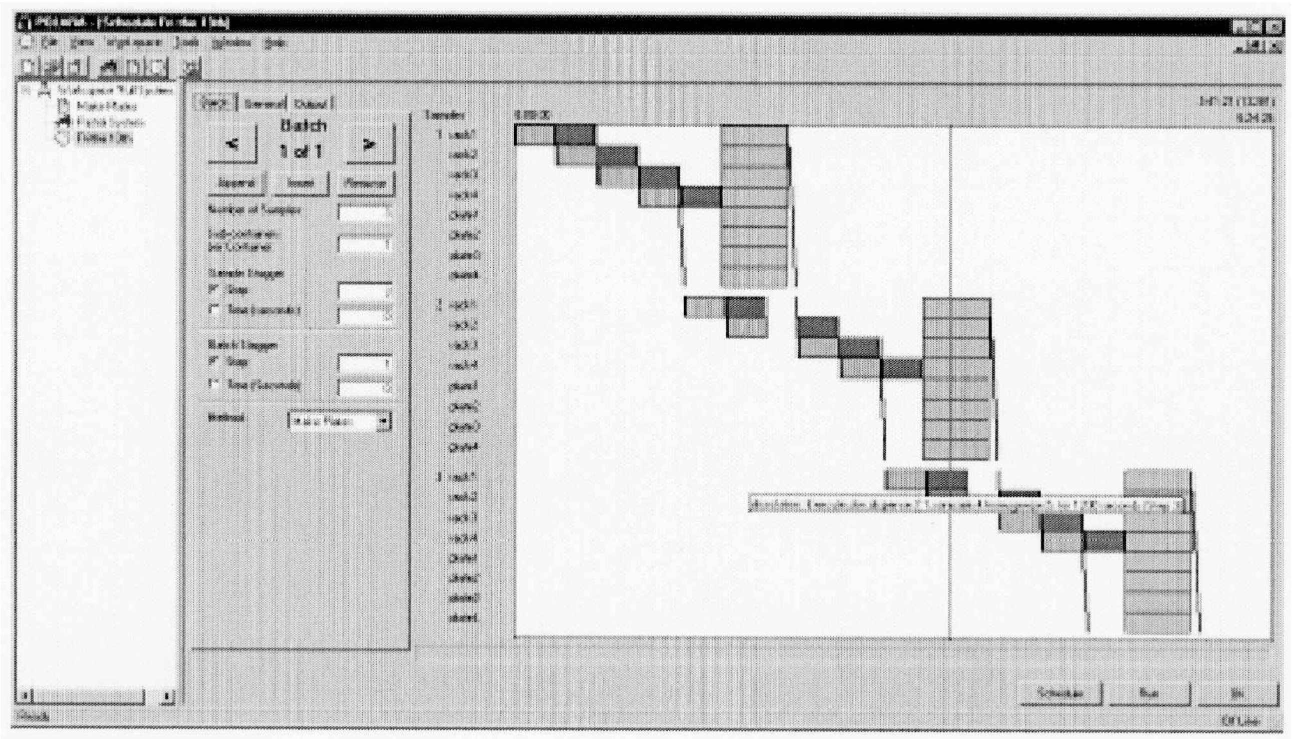

*Figure 5. POLARA schedule view.*

placed racks or plates, programming errors. However, as we got better at setting up runs and the most obvious software bugs were corrected, less frequent but more difficult to solve system problems began to surface. The first one was a design problem with our grippers. We had elected to use a standard microtitre plate gripper from CRS. That gripper has a maximum opening distance of 5.25″ and a minimum closure distance of 3.12″. This is perfectly fine to handle plates in both portrait or landscape orientation. However, our original dual mode gripper used pins on the fingers to mate with holes on the rack for a solid pickup. Clearances and range limitations of the gripper limited the size of the pins and matching holes to about 0.075″. As is now obvious, this was too small to ensure a 0% failure rate in matching up the pins and the holes. Variations in human placement of the racks within the holding plates of the carousel were of the same magnitude as the hole diameter. There were two instances where racks were gripped incorrectly resulting in crashes. A new design using a shelf-like plate has not seen a failure yet. Since clearances in landscape mode are tight, we are replacing the gripper with a new model from CRS that offers a wider maximum opening. After experimenting with various materials for 'pads' for plate pickup (sandpaper, rubbers, etc.), we concluded that sharp pins were the most reliable.

In parallel with system fixes described above, instrument control software for the three Bohdan workstations was rewritten in Visual Basic 6. This allowed for better error handling, faster workstation executions, and tight integration with our databases.

### Plate replication system

The plate replication system (figure 6) had a simple goal: to replicate mother plates into a number of labelled and sealed daughter plates. Because of our experience with the CRS arm and POLARA, we chose to use the same for this system. Even though the payload of the A465 was not required, its long reach (about 34″) made it possible to design a system without a track, in a fairly small footprint of $4' \times 6'$.

The Beckman Multimek 96 was selected as the 96-well pipetter because several were already installed in units in the Company and for its 384 well indexing capability. The CRS carousel allowed for the storage of 96 daughter plates (0.7 ml) with every other shelf removed. The plate labeler selected is the Zymark Presto Labeler. It was chosen for its small footprint and for the availability of an OCX for easy integration. A portion of the cover had to be cut off to allow for gripper clearance during landscape plate loading from the side. The plate sealer is the ALPS 1000 heat sealer. For plate shipments adhesive sealers are not appropriate and cap mats have a tendency to pop off. Heat sealers, however, require plates with chimneys around the wells.

Since this system is fairly self-contained, we integrated it using a single PC. The sealer is controlled directly from the RAPL-3 component of the Sealer 'Interface' via a simple contact closure on a GPIO block. The Multimek is controlled from its own control application which in turn is launched from a small Visual Basic application that communicates with the RAPL-3 component of the POLARA 'Interface' via a named pipe. The Presto Labeler has a small custom Visual Basic application which incorporates the Zymark OCX. This also uses a named pipe to communicate with POLARA.

Mother plates are housed in a simple stand alone hotel (figure 7). A barcode scanner is used to manually scan in the mother plates on the hotel. A Visual Basic application then registers all of the daughter plates and generates the list of barcodes to be applied on the daughter plates. The whole replication process is now taking about 1 minute per plate.

Use of the integrated system has just begun. So far the only source of failure has been with label application. The problem is erratic and difficult to reproduce and hence debug. We are working with Zymark to resolve this problem.

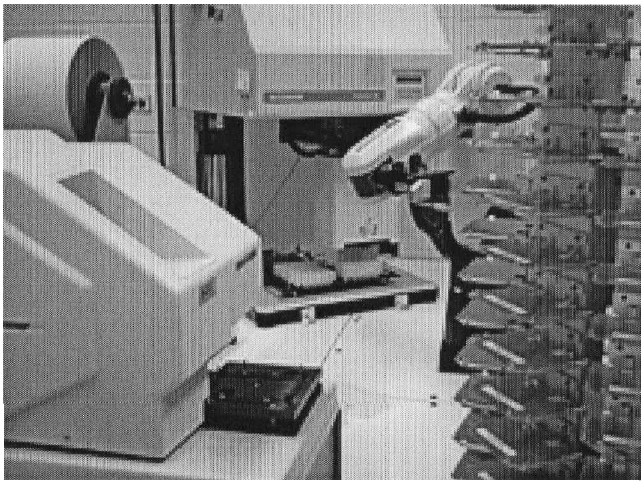

*Figure 6. Plate replication system.*

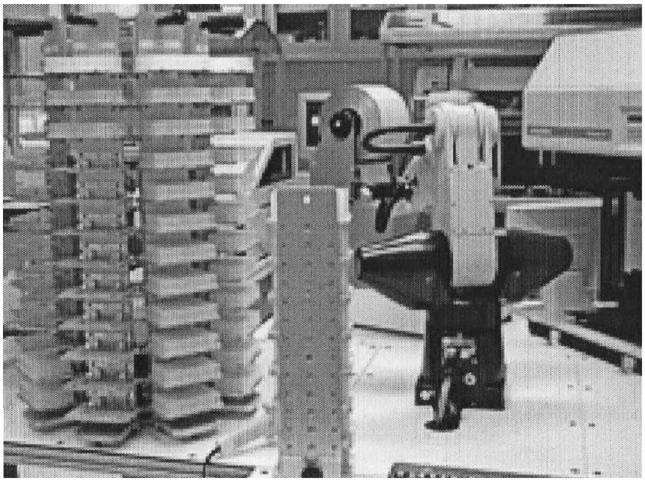

*Figure 7. Plate replication system (side view).*

### Acknowledgements

I wish to acknowledge the very special contribution of Miguel Maccio to the implementation of the first system. Thanks also to Kevin Blake, Gary Kath, Brian Uhrig, from our Bioelectronics Group, Gary Mallow, and Chris Napolitano.

### References

1. Cragg, G. *et al.*, 1997, Natural products in drug discovery and development. *Journal of Natural Productions*, **60**, 52–60.
2. Maccio, M. A., Blake, K., Holt, T. and Doherty, P. J., 1999, Drying Saucer: a 96-well format microplate evaporator. *Journal of the Association for Laboratory Automation*, **4**, 31–35.

